# Distinct Patterns of the Lipid Alterations between Genotype 1 and 2 Chronic Hepatitis C Patients after Viral Clearance

**DOI:** 10.1371/journal.pone.0104783

**Published:** 2014-08-14

**Authors:** Ming-Ling Chang, Yung-Kuan Tsou, Tsung-Hui Hu, Cheng-Hui Lin, Wey-Ran Lin, Chang-Mu Sung, Tsung-Hsing Chen, Mei-Ling Cheng, Kuo-Chin Chang, Cheng-Tang Chiu, Chau-Ting Yeh, Jong-Hwei Su Pang, Ming-Shi Shiao

**Affiliations:** 1 Liver Research Center, Division of Hepatology, Department of Gastroenterology and Hepatology, Chang Gung Memorial Hospital, Taoyuan, Taiwan; 2 Graduate Institute of Clinical Medical Sciences, Chang Gung University, Taoyuan, Taiwan; 3 Division of Hepato-Gastroenterology, Department of Internal Medicine, Kaohsiung Chang Gung Memorial Hospital and Chang Gung University College of Medicine, Kaohsiung, Taiwan; 4 Department of Laboratory Medicine, Chang Gung Memorial Hospital, Linkou, Taiwan; Kaohsiung Medical University Hospital, Kaohsiung Medical University, Taiwan

## Abstract

**Background:**

The hepatitis C virus (HCV) genotype-specific impacts on the host metabolic alterations remained inconclusive.

**Methods:**

A prospective study including 229 (118 genotype 1 (G1) and 111 G2) consecutive chronic HCV patients who had completed a course of anti-HCV treatment and underwent pre- and 24 weeks post-treatment surveys of metabolic profiles was conducted. Patients were stratified according to the therapeutic response, viral genotype and baseline insulin resistance (IR: homeostasis model assessments of IR (HOMA-IR) ≥2.5). Paired t-tests were used to compare the pre- and post-treatment variables.

**Results:**

Significant post-therapeutic increases in cholesterol, triglyceride, HDL, LDL, apolipoprotein A1 and apolipoprotein B were observed in patients with sustained virological response (SVR) but not in those without. Among those with SVR, post-therapeutic increases in HDL (*p<0.001*) and apolipoprotein A1 (*p* = 0.012) were only found in G2, whereas increased triglyceride/HDL (*p* = 0.01) ratios were only found in G1 patients. When stratified by baseline IR among those with SVR, a significant increase in post-treatment HDL (*p* = 0.019) and apolipoprotein A1 (*p* = 0.012) but a decrease in HOMA-IR (*p* = 0.04), C-peptide (*p* = 0.019) and hemoglobin A1c (*p* = 0.047) were found in patients with baseline IR; a significant increase in HOMA-IR (*p* = 0.002) was found in patients without baseline IR. The latter change was observed only in G1 (*p* = 0.01) but not G2 patients. Although the pre-treatment metabolic profiles of G1 and G2 patients were indifferent, G1 had higher post-treatment triglyceride/HDL ratios (*p* = 0.041) and triglyceride (*p* = 0.044) levels than G2 patients.

**Conclusions:**

G2 benefit more than G1 patients from viral clearance in metabolic alterations, particularly in those without baseline IR.

## Introduction

The hepatitis C virus (HCV) is a human pathogen responsible for acute and chronic liver disease, infecting an estimated 130–170 million individuals worldwide [1]. Its variants are classified into six genotypes [2]. In addition to cirrhosis and hepatocellular carcinoma, HCV induces several complications, including steatosis, dyslipidemia and insulin resistance (IR) [3], [4]. Accordingly, It is now considered to cause metabolic alterations instead of being simply a viral infection. In particular, much of the HCV life cycle including the entry into naïve cells, infectivity, RNA replication, viral assembly and viral secretion closely associated with lipid metabolism [1], [3]. The combination of pegylated interferon (Peg-IFN)-α plus ribavirin has provided a ‘cure’ for a considerable proportion of patients with chronic hepatitis C (CHC), particularly when most patients have the favorable interleukin-28B (IL-28B) genotype [5]. In addition to increasing the curability of difficult-to-treat cases, one of the current challenges is to determine the reversibility of the metabolic alterations and associated complications after viral clearance.

Several contradictions have been noted concerning HCV-related metabolic alterations. First, most large-scale case-control studies have demonstrated that HCV infection leads to lower total cholesterol (TC) levels [6], [7]. In a large series, HCV-associated hypocholesterolemia was shown to be most evident with genotype 3 (G3), intermediate with genotype 1 (G1) and not significant with genotype 2 (G2) [8]. However, a proportional relationship was ever reported between TC and the viral load in G2 patients [9]. The source of the apparent conflicts in genotype-specific hypolipidemia of CHC patients may result from individual bias which can not be eliminated completely from case-control studies. Second, the eradication of HCV was regarded to reduce the incidences of type 2 diabetes in both G1 and G2 patients [10]. However, reduced IR after sustained virological responses (SVRs) was observed in G1 but not in G2 or G3 patients [11]. Furthermore, a recent prospective study enrolled G1, 2, 3 and 4 CHC patients failed to demonstrate any difference between the mean pre- and post-anti-HCV treatment homeostasis model assessments of IR (HOMA-IR) values in patients with SVR [12]. The above inconsistencies may arise from the heterogeneous baseline glucose metabolisms of the hosts. Given the inconclusive genotype-specific impact of HCV infection on IR, how IR affects lipid metabolism in CHC patients remains even more unclear.

Comparing the pre- and post-treatment variables in CHC patients with SVR has provided an excellent opportunity to eliminate the interference caused by individual bias when reviewing the impact of HCV on metabolic alterations [12]. Interferon therapy has been associated with increases in TC and triglyceride (TG) levels [13]. However, the impacts of HCV should not be masked by any interferon effect when comparing patients with and without SVR. How viral genotype and baseline glucose metabolism affect the overall host metabolisms might be elucidated by further stratifying the CHC patients by genotype and baseline IR. Therefore, we sought to address the puzzling observations of HCV-related metabolic alterations by conducting a prospective study analyzing the metabolic profiles of CHC patients before and after anti-HCV therapy.

## Materials and Methods

### Patients

The study group consisted of subjects ≥18 years old with CHC identified by documented HCV antibody (HCV Ab) positivity and detectable HCV RNA for more than 24 weeks. Subjects with liver cirrhosis (LC), alcoholic liver disease, human immunodeficiency virus (HIV), hepatitis B infection, hemochromatosis, coronary heart disease, malignancy and recipients of solid organ transplants as well as those on lipid-lowering (statin and fibrate), glucose-lowering (metformin, glipiride, long acting basal insulin analogue and dipeptidyl peptidase-4) or anti-hypertension (calcium channel blocker, β blocker, α1-antagonist, diuretics, angiotensin II receptor antagonist, angiotensin converting enzyme inhibitor and nitroglycerin) medications were excluded. LC was diagnosed by histologic findings or repeated ultrasonographic findings consistent with LC, supplemented with clinical features such as esophageal or gastric varices and thrombocytopenia, as described elsewhere [14]. The nonalcoholic nature of the disease was established by clinical assessment that alcohol consumption was less than 10 g/day for women and 20 g/day for men [15]. Coronary heart disease, malignancy and recipients of solid organ transplants were diagnosed by history and chart review, and further confirmed by specialist while indicated. Diet control and adequate aerobic exercise were recommended for the enrolled patients with glucose or lipid abnormality.

### Study design

118 G1 and 111 G2 Taiwanese patients with CHC consecutively recruited at a tertiary care center between January 2010 and January 2013 were treated with Peg-IFN-α2b (1.5 µg/kg/week, subcutaneously) (PEG-Intron; Schering-Plough Inc., Kenilworth, NJ,USA) and ribavirin (800–1400 mg/day, in two divided doses), according to the reimburse policy of the Bureau of National Health Insurance (BNHI) of the country with some modification. The G1 and the G2 patients received 48-week and 24-week duration of treatment, respectively. All the recruited patients received at least 80% of total Peg-IFN dose, at least 80% of total ribavirin dose and completed at least 80% of total study duration (80/80/80 adherence) [16]. The patients were evaluated for the presence of HCV RNA at 2 weeks before therapy; at 4, 12 and 24 weeks during therapy; at the end of therapy; and 24 weeks after therapy, according to the BNHI's guideline. SVR was defined as undetectable HCV RNA levels at 24 weeks after the completion of therapy. Single-nucleotide polymorphism of *IL28B* (rs12979860) was assessed using genomic DNA isolated from EDTA anti-coagulated peripheral blood from the patients, as previously described [17]. The HCV RNA levels were tested using the COBAS Amplicor (ver. 2.0, Roche Diagnostics, Tokyo, Japan). The HCV genotype was determined using the InoLipa method (COBAS AmpliPrep/COBAS TaqMan HCV Test, Roche Diagnostics, Tokyo, Japan). The lipid panels (TG, low-density lipoprotein-cholesterol (LDL), high-density lipoprotein-cholesterol (HDL), TC, apolipoprotein A1 (Apo AI) and apolipoprotein B (Apo B)), glucose profiles (fasting glucose, insulin, C-peptide, glycated hemoglobin and HOMA-IR ([fasting insulin (µU/mL)×fasting glucose (mmol/L)]/22.5)), aspartate aminotransferase (AST), alanine aminotransferase (ALT), platelet count and HCV core antigen (Ag) assays (Abbott Labs) were evaluated for all patients 2 weeks before and 24 weeks after therapy. The AST to platelet ratio index (APRI) ((AST/34)×100/(platelet count/10^9^/L)) was used to assess liver fibrosis [18]. Body weight and body mass index (BMI) were also determined during each measurements. IR was defined using a HOMA-IR score ≥2.5 [19]. Abdominal ultrasound studies were performed in every patient before therapy and every 6 months afterward to monitor fatty liver, cirrhosis and tumors.

### The primary and secondary objectives

The primary and secondary objectives of the current study were to access the HCV viral genotype-specific impacts on the metabolic alteration of the host and to evaluate the interaction between the glucose and lipid metabolism in the CHC patients, respectively.

### Biochemistry

AST(<34 U/L), ALT(<36 U/L), platelet (150–450×10^9^/L), fasting glucose (Glu (AC): 70–105 mg/dL), insulin (2–17 µIU/mL), C-peptide (0.9–4.3 ng/mL), glycated hemoglobin (hemoglobin A1c) (HbA1c (4.6–5.6%; 27–38 mmol/mol), lipid profile (TC: <200 mg/dL; TG: <150 mg/dL; HDL: male (M): >40 mg/dL; female (F): >50 mg/dL; LDL: <100 mg/dL), Apo AI (M: 1.10–2.05 g/L; F: 1.25–2.15 g/L), Apo B (M: 0.55–1.4 g/L; F: 0.55–1.25 g/L), HCV core Ag (≥3.00 (reactive) (fmol/L)), hepatitis B virus surface antigens (Ag), HCV Ab (AxSYM 3.0; Abbott Laboratories, Chicago, IL, USA), HIV Ag/Ab combi test, transferrin saturation (M: 15–50%; F: 12–45%), ferritin (M: 20–290 ng/mL; F: premenopausal 4.5–170 ng/mL, postmenopausal 24–260 ng/mL**)**, and HCV RNA (1.5×10^−5^∼6.9×10^7^ IU/ml) were measured in the clinical pathology or liver research laboratories of the hospital using routine automated techniques.

### Statistics

All statistical analyses were performed using either the Statistical Product and Service Solutions (SPSS ver. 18.0, SPSS Inc. Chicago, USA), MedCalc (MedCalc ver. 12.4, MedCalc Software corp. Maine, USA) or G-power (web-based ver. 3.1) software. The continuous variables were summarized as the means ± standard deviation (SD), and the categorical variables were summarized as the frequencies and percentages. The study size was determined according to reported differences in pre- and post-treatment paired cholesterol levels (mean: 19.7, SD: 19.2, α = 0.05, power = 0.9) [20] with some modification for potential ethnic and viral genotype biases. Univariate linear regression models were used to assess the relationships between various factors to the pre-treatment profiles. The variables found to be associated with the dependent variables in univariate analyses were included in multivariate regression models. The correlations and interactions between the variables as well as confounding factors were determined using correlation and linear regression tests. For a better clarification, the HCVRNA level was log-transformed while indicated. To compare the different variables in the different groups, the continuous variables were analyzed using Student’s t-test, while the categorical variables were analyzed using a chi-squared or Fisher’s exact test, as appropriate. A paired t-test was used with the same variables before and after therapy in the same individuals. Statistical significance was defined at the 5% level based on a two-tailed test of the null hypothesis.

The study protocol conformed to the ethical guidelines of the 1975 Declaration of Helsinki and was approved by the institutional review board of the Chang Gung Memorial Hospital. All the participants had provided their written informed consent to participate in this study.

## Results

### Baseline characteristics of the CHC patients and factors affected pre-treatment HCV RNA levels

The baseline characteristics of the CHC patients were listed in table 1. Total 31 subjects under lipid-lowering (6 subjects), glucose-lowering (12 subjects) or anti-hypertension medications (26 subjects) had been excluded from the study. The pre-treatment HCV RNA and HCV core Ag levels in G1 patients were significantly higher than those of G2 patients, while the SVR rate was higher in the G2 patients relative to the G1 patients. No correlation was found between the HCV core Ag and any of the lipid profile items.

**Table 1:** Baseline characteristics of the CHC patients before anti-HCV treatment.

	G1(*n* = 118)	G2(*n* = 111)	Student’s *t*-test *or chi-squared* *P* values
Male (%)	70 (63.63%)	56 (52.33%)	0.475
Age (yr)#	53.1+/−12.6	55.0+/−11.8	0.251
BMI	24.0+/−3.4	24.9+/−3.4	0.072
HCV RNA (millionIU/ml)	5.1+/−8.3	1.5+/−2.3	<0.001*
Log HCVRNA (IU/ml)	6.10+/−0.98	5.90+/−0.92	0.049*
HCV core Ag (fmol/L)	7643.5+/−6965.2	3497.7+/−3879.4	0.002*
IL-28B rs12979860 CC/CT+TT, n(CC%)#	103/15(87.3%)	104/7(93.6%)	0.595
ALT (U/L)	100.6+/−79.3	112.5+/−82.7	0.354
Fatty liver#	52 (44%)	53 (48%)	0.881
APRI	1.53+/−2.2	1.42+/−1.66	0.817
TG (mg/dL)	108.4+/−59.	99.9+/−44.8	0.259
TC (mg/dL)	170.5+/−32.0	166.9+/−32.2	0.417
HDL (mg/dL)	48.8+/−15.6	48.9+/13.2	0.946
LDL (mg/dL)	103.6+/−40.8	100.5+/−26.1	0.53
TG/HDL	2.62+/−2.35	2.22+/−1.40	0.151
Apo AI (g/L)	1.57+/−1.09	1.38+/−0.25	0.166
Apo B (g/L)	0.83+/−0.20	0.81+/−0.19	0.651
Glucose (AC) (mg/dL)	100.8+/−33.0	100.7+/−29.9	0.990
Insulin (µIU/mL)	14.19+/−22.34	10.61+/−10.34	0.2
C-peptide (ng/mL)	2.93+/−3.02	2.76+/−2.09	0.712
Hb-A1c (%; mmol/mol)	5.90+/−1.02; 41+/−11	5.75+/−0.75; 39.5+/−8.5	0.317
HOMA-IR	4.25+/−10.33	2.67+/−3.01	0.19
SVR (%)#	90 (77.5%)	100 (91.7%)	0.005*

# chi-squared test;

*p<0.05.

### Differences between those with and without SVR: increases in all lipids were noted among the patients with SVR, but not in those without

Increases in the TG, TC, HDL, LDL, Apo AI and Apo B levels were found in patients with SVR but not in those without. The glucose profile levels were not different, regardless of the therapeutic response (Table 2).

**Table 2:** Comparison of pre- and post-treatment variables in the CHC patients stratified by therapeutic response.

	SVR (+)(*n* = 195)		Paired *t*-test*P* values	SVR (−)(*n* = 34)		Paired *t*-test*P* values
	Pre- treatmentvalue	Post-treatmentvalue		Pre-treatmentvalue	Post-treatment value	
Male (%)	109 (60%)			16 (52%)		
Age (yr)	53.7+/−12.4			56.9+/−11.4		
BMI	24.4+/−3.4	24.2+/−3.4	0.65	23.9+/−3.3	22.1+/−5.9	0.099
HCV RNA(millionIU/ml)	2.9+/−6.5	0	<0.001*	5.8+/−5.2	3.0+/−5.4	0.035*
Log HCV RNA(IU/ml)	5.94+/−1.01			6.48+/−0.48	6.09+/−0.70	0.168
IL-28B rs12979860CC/CT+TT, n(CC%)	183/11(93.8%)			23/11(68.75%)		
TG (mg/dL)	100.3+/−45.5	119.3+/−72.8	<0.001*	127.4+/−85.5	109.8+/−45.0	0.134
TC (mg/dL)	167.4+/−31.5	183.0+/−37.2	<0.001*	179.1+/−26.9	181.6+/−36.4	0.700
HDL (mg/dL)	48.5+/−13.7	50.7+/−13.8	0.006*	53.2+/−18.4	53.8+/−16.7	0.740
LDL (mg/dL)	100.3+/−27.4	112.1+/−32.6	<0.001*	105.3+/−24.5	108.7+/−34.0	0.584
TG/HDL	2.32+/−1.53	2.77+/−2.58	0.006*	2.93+/−3.76	2.24+/−1.66	0.151
Apo AI (g/L)	1.40+/−0.26	1.44+/−0.25	0.006*	1.46+/−0.25	1.45+/−0.28	0.818
Apo B (g/L)	0.82+/−0.19	0.88+/−0.21	<0.001*	0.93+/−0.26	0.86+/−0.33	0.128
Glucose (mg/dL)	99.5+/−30.2	103.8+/−48.8	0.117	109.9+/−40.1	100.2+/−36.0	0.238
Insulin (µIU/mL)	11.51+/−14.76	11.26+/−17.19	0.783	19.58+/−30.01	23.32+/−45.06	0.421
C-peptide (ng/mL)	2.61+/−1.92	10.75+/−83.87	0.329	4.94+/−5.52	3.69+/−2.80	0.147
Hb-A1c (%; mmol/mol)	5.82+/−0.91;40+/−10	5.70+/−0.85;38.5+/−9.5	0.125	5.91+/−0.91;41+/−10	6.01+/−1.14;42.5+/−12.5	0.493
HOMA-IR	3.14+/−7.14	2.84+/−4.20	0.407	5.77+/−9.83	6.66+/−13.48	0.491

*p<0.05.

### Differences between G1 and G2 patients after SVR: G2 patients demonstrated increased HDL and Apo AI levels, whereas G1 patients demonstrated increased TG/HDL ratios

Among the patients with SVR, only G2 patients displayed increased post-treatment HDL and Apo AI levels. In contrast, G1 patients exhibited increased post-treatment TG/HDL ratios (Table 3). Neither G1 nor G2 patients had significantly different post-treatment HOMA-IR compared to their pre-treatment levels.

**Table 3:** Comparison of pre- and post-treatment variables in the CHC patients with SVR stratified by genotype.

	G1 with SVR(*n* = 93)		Paired *t*-test*P* values	G2 with SVR(*n* = 102)		Paired *t*-test*P* values
	Pre-treatmentvalue	Post-treatmentvalue		Pre-treatmentvalue	Post-treatmentvalue	
Male (%)	56 (65.4%)			55 (56.5%)		
Age (yr)	52.0+/−13.2			55.0+/−12.6		
BMI	24.0+/−3.4	24.0+/−3.7	0.323	24.7+/−3.7	24.5+/−3.4	0.17
IL-28B rs12979860CC/CT+TT, n(CC%)	89 (95.6%)			95 (93.18%)		
TG(mg/dL)	105.1+/−50.2	134.5+/−84.4	0.001*	96.6+/−40.2	108.3+/−64.4	0.017*
TC(mg/dL)	168.3+/−32.3	182.5+/−37.3	<0.001*	165.8+/−31.1	183.1+/−37.5	<0.001*
HDL(mg/dL)	48.1+/−14.9	48.9+/−13.5	0.478	48.6+/−12.9	51.7+/−14.2	0.001*
LDL(mg/dL)	100.4+/−28.9	109.7+/−32.8	0.01*	100.0+/−26.4	113.5+/−32.9	<0.001*
TG/HDL	2.4+/−1.6	3.2+/−3.1	0.01*	2.2+/−1.4	2.3+/−1.9	0.308
Apo AI(g/L)	1.43+/−0.28	1.45+/−0.24	0.361	1.38+/−0.24	1.44+/−0.26	0.012*
Apo B (g/L)	0.81+/−0.18	0.89+/−0.20	0.001*	0.81+/−0.19	0.85+/−0.21	0.019*
Sugar (mg/dL)	99.1+/−32.3	104.4+/−64.3	0.324	99.6+/−28.5	103.2+/−30.1	0.099
Insulin(µIU/mL)	12.8+/−18.9	13.8+/−25.1	0.487	10.4+/−10.4	9.2+/−5.5	0.333
C-peptide (ng/mL)	2.5+/−1.6	2.7+/−2.2	0.22	2.7+/−2.1	2.1+/−1.2	0.032*
Hb-A1c(%; mmol/mol)	5.9+/−1.0;41+/−11	5.7+/−0.6;38.5+/−6.5	0.081	5.7+/−0.7;38.5+/−7.5	5.6+/−1.0;38+/−11	0.413
HOMA-IR	3.8+/−10.2	3.3+/−5.8	0.475	2.6+/−3.0	2.4+/−2.2	0.698

*p<0.05.

Additionally, G1 patients exhibited significantly higher post-treatment TG levels (134.56±84.46 vs. 108.38±64.45 mg/dL, *p* = 0.032) and TG/HDL ratios (3.27±3.11 vs. 2.34±1.95, *p* = 0.037) than G2 patients.

### Differences between those with and without baseline IR after SVR: increases in Apo AI and HDL levels but decreases in HOMA-IR, C-peptide and HbA1c levels were noted in patients with baseline IR, but not in those without

Among those with SVR, patients with baseline IR exhibited increased post-treatment lipid profiles, including the HDL and Apo AI levels (Table 4). However, no reversal of Apo B was observed. Moreover, some glucose profiles, including the levels of HOMA-IR, C-peptide and HbA1c, decreased. In contrast, in patients without baseline IR, Apo B rather than Apo AI levels increased; furthermore, glucose, insulin and HOMA-IR increased after SVR. However, none of the post-treatment glucose profiles reached the criteria for diabetes or IR.

**Table 4:** Comparison of pre- and post-treatment variables in the CHC patients with SVR stratified by baseline IR.

	SVR (G1 and G2) with baseline IR(*n* = 63)		Paired *t*-test*P* values	SVR (G1 and G2) without baseline IR(*n* = 132)		Paired *t*-test*P* values
	Pre- treatmentvalue	Post-treatmentvalue		Pre- treatmentvalue	Post-treatmentvalue	
Male (%)	37 (62.7%)			68 (60.1%)		
Age (yr)	54.5+/−12.6			52.3+/−12.5		
BMI	25.7+/−3.6	25.5+/−3.5	0.111	23.5+/−3.0	23.3+/−2.9	0.201
IL-28B rs12979860CC/CT+TT, n(CC%)	59/4(93.6%)			121/7(91.66%)		
TG(mg/dL)	106.1+/−45.0	130.6+/−85.1	0.004*	96.0+/−44.7	113.1+/−65.5	0.003*
TC(mg/dL)	166. 0+/−29.7	182.4+/−40.9	<0.001*	168.3+/−32.8	184.1+/−36.1	<0.001*
HDL(mg/dL)	43.8+/−13.2	47.0+/−11.6	0.019*	51.2+/−13.6	52.4+/−14.7	0.188
LDL(mg/dL)	100.5+/−25.5	112.3+/−35.9	0.002*	100.3+/−28.6	112.9+/−31.4	<0.001*
TG/HDL	2.7+/−1.8	3.2+/−3.0	0.135	2.0+/−1.2	2.4+/−2.2	0.022*
Apo AI(g/L)	1.28+/−0.26	1.37+/−0.23	0.012*	1.45+/−0.26	1.48+/−0.26	0.111
Apo B (g/L)	0.84+/−0.17	0.87+/−0.20	0.274	0.80+/−0.19	0.88+/−0.23	<0.001*
Sugar (mg/dL)	116.3+/−44.6	120.1+/−76.5	0.601	90.2+/−8.9	95.0+/−15.7	0.001*
Insulin (µIU/mL)	19.7+/−21.9	16.8+/−27.0	0.223	6.9+/−2.8	8.0+/−4.6	0.016*
C-peptide (ng/mL)	3.9+/−2.5	3.0+/−1.5	0.019*	1.9+/−1.0	2.2+/−1.8	0.095
Hb-A1c (%; mmol/mol)	6.2+/−1.3;44+/−14	5.9+/−0.8;41+/−9	0.047*	5.6+/−0.4;37.5+/−4.5	5.6+/−0.5;37.5+/−5.5	0.546
HOMA-IR	6.2+/−11.7	4.6+/−6.7	0.04*	1.5+/−0.5	1.9+/−1.2	0.002*

*p<0.05.

### Differences between G1 and G2 patients without baseline IR after SVR: G2 patients demonstrated increased HDL and Apo AI levels, while G1 patients demonstrated increased TG levels, TG/HDL ratios and HOMA-IR levels

**Figure 1:**
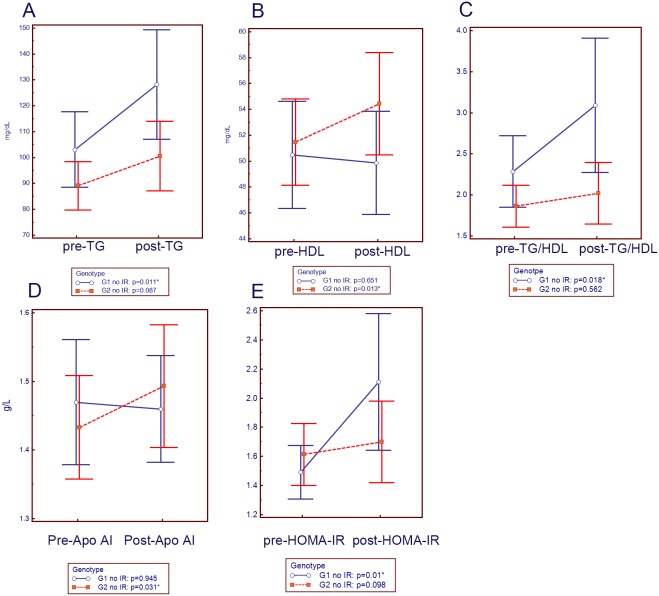
Comparisons of the pre- and post-treatment variables when using paired-t tests. Pre-: pre-treatment; post-: post-treatment; G1 no IR: G1 CHC patients without baseline IR achieved SVR (n = 65); G2 no IR: G2 CHC patients without baseline IR achieved SVR (n = 67). *: *p*<0.05.

Among the G1 (n = 65) and G2 (n = 67) patients without baseline IR who achieved SVR (Figure 1), G2 patients showed increased HDL and Apo AI levels, while G1 patients exhibited increased TG levels, TG/HDL ratios and HOMA-IR levels. However, the increased post-treatment HOMA-IR in G1 patients did not reach the criteria for IR or diabetes. Besides, G1 patients had significantly higher post-treatment TG levels (130.89±73.73 vs. 100.88±51.82 mg/dL, *p* = 0.044) and TG/HDL ratios (3.11±2.84, vs. 2.01±1.42, *p* = 0.041) than G2 patients.

## Discussion

The most compelling findings of the current study are as follows: (1) Although the HCV viral clearance in both G1 and G2 patients resulted in increases in most lipid profile items, post-therapeutic increases in HDL and Apo AI levels were found only in G2 patients; increased TG/HDL ratios were found only in G1 patients. Moreover, after SVR, G2 patients had lower post-treatment TG/HDL ratios and TG levels than G1 patients. (2) Among the patients without baseline IR, G1 patients had increased post-treatment HOMA-IR levels, in contrast to G2 patients. (3) Among the patients with baseline IR, part of the lipid profile including Apo AI and HDL levels increased and part of glucose profile including HOMA-IR, C-peptide and HbA1c levels decreased significantly after viral clearance.

The prevalences of G1 CHC increased with age, while that of G2 CHC decreased with age [21]. However, in the current and other studies enrolled CHC patients consecutively [22], except pre-treatment HCV RNA, no other baseline characteristics, including age, were different between the G1 and G2 patients. Besides, in white patients with G1 CHC, the favorable IL28B rs12979860 CC genotype is associated with less pronounced disturbances in lipid metabolism [23] and with reduced IR [24]. In the current study, however, over 90% of the CHC patients with SVR had favorable IL-28B genotype regardless of HCV genotype (Table 3). Therefore, host factors were less likely to explain the various metabolic alterations in the G1 and G2 patients after viral clearance. The HCV core Ag is strongly associated with serum lipoviral particles [25]. However, it did not correlate with any of the pre-treatment lipid profile items. Individual variation might account for this discrepancy. This observation highlights the importance of examining the changes in the lipid profiles after SVR in the same individuals.

The current study demonstrates that G2 clearance affected the lipid profile more favorably than clearing G1 HCV did. Apart from TC, LDL, TG, and Apo B, G2 HCV clearance also increased the HDL and Apo AI levels, while G1 viral clearance increased the TG/HDL ratios (Table 3). After SVR, the G1 patients had higher post-treatment TG levels and TG/HDL ratios than the G2 CHC patients. These genotype-specific impacts on metabolic profiles were even more evident after eliminating the influence of IR, as G1 but not G2 had increased post-therapeutic TG and HOMA-IR levels (Figure 1). Because high TG, low HDL levels and high TG/HDL ratios all indicate a metabolic syndrome, a cluster of cardiovascular risk factors due to IR [26], [27], the worse metabolic profile after SVR might potentially lead to higher cardiovascular risks in G1 patients, as compared with G2 patients. This distinct pattern of lipid alterations between the G1 and G2 CHC patients after SVR is particularly important in areas like Taiwan, where most HCV infections are either G1 or G2 [21], [22]. Although the reversal of hypolipidemia in patients with CHC after SVR has been documented in previous studies [20], [28]–[30], to the best our knowledge, this is the first clinical study to disclose the genotype-specific impacts from G1 and G2 HCV infection on lipid metabolism. During basic research, G2 exhibited a significantly higher HDL binding rate than G1 HCV did [31]. After SVR, the higher levels of “free” HDL relieved from the G2 relative to the G1 HCV complex may contribute to the increased HDL in the G2 patients. Whether there are any differences in the composition of HDL between the G1 and G2 patients before or after SVR requires further investigations like lipoprotein electrophoresis. Furthermore, significantly different viral load of G1 and G2 HCV might have led to different liver damage and subsequently different lipoprotein metabolism.

Among cases without baseline IR, after SVR, increased HOMA-IR levels were noted but did not reach the threshold for diabetes or IR, possibly indicating negligible biological importance (Table 4). It may arise directly from a less favorable lipid rather than glucose metabolism. In contrast, among patients with baseline IR, not only HOMA-IR, but also C-peptide and HbA1c levels decreased after SVR. It was compatible with previous studies [16], [32]. Also, because IR usually decreases Apo AI and HDL levels [33], the increases in Apo AI and HDL levels in those with baseline IR might stem directly from improved glucose metabolism. Therefore, SVR seemed to give rise to favorable glucose and subsequent lipid alterations in CHC patients with baseline IR. The clinical evidence also supports the finding since a large cohort study had shown the improved cardiovascular outcomes in diabetic CHC patients after anti-HCV treatment [34].

However, the patients under lipid-lowering, glucose-lowering or anti-hypertension medications were excluded in the current study. It implied that those with severe metabolic disease were not counted in the longitudinal analysis. Besides, all of the comparisons in the profiles between G1 and G2 patients were cross-sectional. A large-scale, prospective, randomized-controlled study with minimal exclusion criteria might be indicated to verify the genotype-specific metabolic alteration among those with extremely severe metabolic disease.

The major limitation of the current study was the lack of biopsies to validate the hepatic steatosis and fibrosis. We used ultrasonographic finding to diagnose hepatic steatosis, which is defined as the presence of hepatic steatosis in ≥5% of hepatocytes [35]. Although ultrasonography has 60–94% sensitivity and 84–95% specificity for detecting hepatic steatosis [36], its sensitivity is reduced when <30% of the liver parenchyma is infiltrated by fat [36]. Thus, ultrasonography might underestimate the prevalence of hepatic steatosis. The real role of steatosis in the genotype-specific impacts might need further verifications using liver biopsy or magnetic resonance spectroscopy [37]. To assess hepatic fibrosis, APRI is a noninvasive alternative to liver biopsies, particularly for HCV infections [18]. Whereas an updated meta-analysis had demonstrated that APRI may be only reliable in detecting severe fibrosis [38]. No difference in the pre-treatment APRI levels were found between the G1 and G2 patients might only explain the minimal role of severe fibrosis in the genotype-specific metabolic alterations. To explore the impact of mild fibrosis, liver biopsy is still demanded.

Despite the favorable lipid profile, HCV infections increase cardiovascular events and associated mortalities in large-scale studies [39], [40]. Several population-based cohort studies had shown that anti-HCV treatment is associated with improved the long-term cardiovascular outcomes in the CHC patients [34], [41]. However, reversing hypolipidemia after SVR may further the emergence of cardiovascular disease in extreme cases [42]. Whether all CHC patients benefit equally from SVR regarding cardiovascular risk must be determined. The current study demonstrated that G2 patients may benefit more than G1 patients from viral clearance in metabolic alterations, particularly in those without baseline IR. Therefore, after SVR, the follow-up protocols used to survey cardiovascular events might be individualized in CHC patients according to their viral genotype and the baseline glucose metabolism, at least within 24 weeks after completion of anti-HCV therapy.
